# Barriers to effective communication in UAE community pharmacies: general public perspectives on enhancing patient-pharmacist interaction and policy development

**DOI:** 10.1080/20523211.2025.2460744

**Published:** 2025-02-13

**Authors:** Zelal Kharaba, Ahmad Al-Azayzih, Sayer Al-Azzam, Anan Jarab, Hisham E. Hasan, Dania Rahhal, Feras Jirjees, Khalid A. Al-Kubaisi, Monia H. Aljumah, Faris El-Dahiyat, Sara Meer, Mohammad A. Y. Alqudah, Mamoon A. Aldeyab, Karem H. Alzoubi

**Affiliations:** a Department of Pharmacy Practice and Pharmacotherapeutics, College of Pharmacy, University of Sharjah, Sharjah, United Arab Emirates; b Faculty of Medical Sciences, Newcastle University, Newcastle upon Tyne, UK; c Department of Clinical Pharmacy, Faculty of Pharmacy, Jordan University of Science and Technology, Irbid, Jordan; d Department of Clinical Pharmacy, College of Pharmacy, Al Ain University, Abu Dhabi, United Arab Emirates; e Department of Pharmacy, School of Applied Sciences, University of Huddersfield, Huddersfield, UK

**Keywords:** Community pharmacy, effective communication, United Arab Emirates, pharmacy services, environmental barriers, personal barriers

## Abstract

**Background:**

Community pharmacies serve as accessible points of care, offering essential services such as medication dispensing, health consultations, vaccinations, and chronic disease management, thereby playing a critical role in the healthcare system. This study aims to identify and evaluate the factors that obstruct general public-pharmacist interactions, providing insights to enhance pharmacy environments.

**Methods:**

A total of 406 general publics were surveyed using a validated questionnaire. Statistical analysis was performed with categorical variables presented as frequencies and percentages, and continuous variables as medians with 95% confidence intervals. Binary regression models were used to explore the relationships between sociodemographic characteristics and communication barriers, with a significance level set at *p* < 0.05 for all analyses.

**Results:**

The primary reasons for pharmacy visits were collecting prescription medications (61.58%) and purchasing OTC products (55.17%), while health screenings were less common (9.11%). Barriers to effective communication included crowded environments (61.58%), limited counseling time (45.81%), and lack of privacy (45.07%). Binary logistic regression revealed that participants who visited the pharmacy weekly were 5.5 times more likely to experience high environmental barriers (OR: 5.502, *p* = 0.002), while interacting with younger pharmacists increased the likelihood of personal barriers (OR: 12.357, *p* = 0.025). Pharmacy proximity (75.12%) and shorter waiting times (47.29%) were the most influential factors in pharmacy preference, while additional services had minimal impact (20.2%).

**Conclusion:**

Effective communication is crucial in community pharmacies for high-quality healthcare. This study identifies key barriers in the UAE and offers insights into targeted interventions to improve communication and public satisfaction.

## Background

Community pharmacies serve as essential components of healthcare systems globally, extending their services beyond medication dispensing to include consultations, vaccinations, and health screenings (Goode et al., 2019; Shirdel et al., 2021). Community pharmacies in the UAE play a key role in the healthcare system, offering services beyond medication dispensing, including health consultations, vaccinations, medication therapy management, and health screenings. Pharmacists are actively involved in patient education, chronic disease management, and promoting healthy lifestyles. As accessible points of care, pharmacies often serve as the first healthcare contact for individuals seeking health advice. Their integration into primary care teams underscores their significance in improving patient outcomes and enhancing public health in the UAE (Kharaba, Farhat, et al., 2022).

However, various barriers, both environmental and personal, can impede effective communication and service delivery within these settings (Al-Azayzih et al., 2023). Effective communication is defined as the exchange of accurate, timely, and comprehensible information between pharmacists and patients, fostering mutual understanding, trust, and collaborative decision-making (Al-Azayzih et al., 2023; Druica et al., 2021). This concept extends beyond basic communication to encompass listening skills, empathy, and patient engagement, distinguishing it from general communication, which focuses on the mere transfer of information, and from patient-centered communication, which emphasises tailoring interactions to the specific needs, preferences, and cultural contexts of patients. Factors influencing pharmacy selection range from proximity and pharmacist knowledge to socioeconomic characteristics like gender, marital status, income, and education level, all of which can impact customers’ experiences and perceptions of communication barriers (Al-Azayzih et al., 2023; Druica et al., 2021).

Effective communication serves as the cornerstone of quality healthcare delivery within community pharmacies services, facilitating the exchange of information, fostering mutual understanding, and promoting patient engagement in decision-making processes (Sharkiya, 2023; Sheehan et al., 2021). In the United Arab Emirates (UAE), community pharmacies play an increasingly prominent role in the healthcare landscape, serving as accessible points of care for individuals seeking health-related guidance and support (Kharaba, Farhat, et al., 2022). While the importance of effective communication in community pharmacy settings is widely acknowledged, there exists a notable gap in the literature regarding the barriers to communication from the perspective of patients in the UAE. While the importance of effective communication in community pharmacy settings is widely acknowledged, there is a notable gap in the literature regarding the barriers to communication from the perspective of patients in the UAE. Although several studies have examined communication barriers in various healthcare settings (Al-Azayzih et al., 2023; Dagnachew et al., 2021), limited research has specifically addressed these barriers within the context of community pharmacies in the UAE, particularly from the patients’ viewpoint. This gap highlights the need for further investigation into the unique challenges faced by patients in this setting to enhance patient-pharmacist communication and improve healthcare delivery in the region.

The pharmaceutical profession has undergone significant evolution, with community pharmacists now recognized as primary care providers who offer advice and manage chronic conditions (Said et al., 2022). Despite their crucial role, challenges such as perceived de-professionalization due to policy changes, online pharmacies, and competition from supermarket drug sales pose threats to their status Factors like loss of professional autonomy, standardization of services, and reduced patient-pharmacist interaction contribute to this decline (Ilardo & Speciale, 2020). Maintaining a focus on patient-centered communication and enhancing professional training are crucial for the future of community pharmacy.

The process of ‘re-professionalization' in pharmacy emphasises the importance of effective communication and patient-centered care. Pharmacists must develop strong communication skills to build trust and engage patients in their treatment decisions, considering factors such as health literacy, cultural backgrounds, and preferences (Ilardo & Speciale, 2020; Kerr et al., 2021; Mort et al., 2010).

Despite efforts to improve accessibility, barriers to healthcare persist for individuals with disabilities, including challenges related to transportation, physical layout, communication, and medication pricing (Clemente et al., 2022; Jairoun et al., 2022). Communication barriers compound difficulties for individuals with hearing and visual disabilities, exacerbating health disparities (Clemente et al., 2022; Jairoun et al., 2022). In Qatar, where the pharmacy profession is experiencing growth, challenges such as limited pharmacist-patient interaction and lack of public awareness of pharmacist services persist, highlighting the need for improved communication strategies and integration into the healthcare system (Kheir & Fahey, 2011).

Given the importance of effective communication in community pharmacy settings, this study aims to explore barriers to communication among patients in the UAE, shedding light on potential areas for improvement and informing strategies to enhance patient-pharmacist interactions.

## Methods

### Study design

The study employed a cross-sectional exploratory survey design, adhering to the Strengthening the Reporting of Observational Studies in Epidemiology (STROBE) guidelines for reporting observational cross-sectional studies (Vandenbroucke et al., 2007). This design allowed for the collection of data at a single point in time, providing insights into the barriers to effective communication in community pharmacies from the perspective of the general public.

### Sampling technique

Participants were recruited from the general public residing in Abu Dhabi, Dubai, Sharjah, Ajman, and other cities across the UAE. Data collection took place over a two-month period, from 15 December 2023, to February 20th, 2024. Convenience sampling was utilised to select participants, aiming for a recommended sample size of 384 individuals calculated using the Raosoft calculator (Raosoft, Inc. 2004). This sample size estimation was based on an assumed population of 9.9 million UAE population, with a 5% margin of error, a response distribution of 50%, and a 95% confidence level estimate for the proportion. Our study successfully recruited 406 participants, exceeding the minimum required sample size, thus making the total number of participants acceptable for analysis.

## Ethical approval

The study obtained ethical approval from the Research Ethics Committee at Al Ain University, with the approval number COP/AREC/AD/18.dated 27 November, 2023. All participants gave their electronic informed consent and took part voluntarily. No identifiable data were collected to ensure participant anonymity.

### Study tool, validation, and reliability

The survey questionnaire was designed following an extensive literature review to identify common physical and personal barriers encountered by the public in community pharmacies (Al-Azayzih et al., 2023; Dagnachew et al., 2021; El Hajj et al., 2011; Ilardo & Speciale, 2020; Jarab et al., 2024; Nsengimana et al., 2022). The questionnaire was carefully structured using clear and accessible language to accommodate the diverse educational and cultural backgrounds of the participants. Validation and reliability assessments were conducted through expert evaluation and a pilot study.

The questionnaire was developed in English and translated into Arabic following a rigorous translation process. This process involved forward and backward translation by two bilingual researchers to ensure the questionnaires’ accuracy.

The survey was structured into five main domains. The first domain assessed the demographic and socioeconomic characteristics of participants and the pharmacy they visited. This includes age, gender, educational level, if they have a chronic disease and overall sociodemographic status of the participants as well as the type of the pharmacy (chain or independent), its location, the approximate number of customers who seek medical advice for the pharmacy. The second domain focused on most common reasons lead the patients to visit a pharmacy including collection of the prescribed/OTC medication or consultation services or purchasing a medical devices or other reasons. The third domain is identifying potential physical and environmental barriers that could impact the quality of pharmaceutical services provided by community pharmacies such as the availability of private counseling areas, the pharmacy’s design and layout, the crowdedness of the dispensing area, the height of the dispensing counter, the comfort and availability of the waiting area, as well as visual and hearing accessibility within the pharmacy. It also considered accessibility for patients with special needs, including elderly and disabled individuals.

The fourth domain explored personal barriers related to the pharmacist's personality, which may impede the provision of optimal services. This section comprised questions that assessed the pharmacist’s confidence during consultations, approachability, knowledge, willingness to address medication-related inquiries, and the ease of discussing sensitive health issues with the pharmacist. It also examined instances where patients may need to communicate with the pharmacist through third parties, such as pharmacy technicians or other staff members.

Finally, the fifth domain investigated the factors influencing patients’ preferences for visiting specific pharmacies, as well as the primary reasons they seek consultation at community pharmacies.

The third, fourth, and fifth domains of the questionnaire were adapted from existing validated tools in the literature (Al-Azayzih et al., 2023; Dagnachew et al., 2021; El Hajj et al., 2011; Ilardo & Speciale, 2020; Jarab et al., 2024; Nsengimana et al., 2022) with minor modifications to ensure cultural relevance and suitability for the UAE context. These adjustments involved refining the language, examples, and context-specific elements to better reflect the healthcare environment and patient population in the UAE.

### Face and content validity

The face and content validity of the questionnaire were established through expert evaluation. A panel of three clinical pharmacy experts, including professors with extensive experience in community pharmacy, and three members of the general public in the UAE, reviewed the items. The experts rated the clarity, relevance, and appropriateness of each question on a scale from 1 to 10 during a virtual session. The mean scores for clarity, relevance, and appropriateness were 8.3, 8.5, and 8.7, respectively. Based on the feedback provided by the panel, necessary revisions were made to enhance the content validity, ensuring that the questions accurately reflected the barriers to communication in community pharmacies in the UAE.

### Reliability

The reliability of the questionnaire was assessed through a pilot test conducted with 15 participants from the general public. During the pilot, participants were asked to complete the questionnaire and provide feedback on any items that were unclear or difficult to understand. To evaluate internal consistency, we calculated Cronbach's alpha for the two main domains: environmental barriers and personal barriers. The Cronbach's alpha values were 0.78 for environmental barriers and 0.72 for personal barriers, indicating an acceptable level of reliability.

### Conceptualisation and operationalisation of barriers

Environmental barriers were conceptualised as physical and organisational factors within the pharmacy that may hinder effective communication and service delivery. These include elements such as the pharmacy layout, availability of private counseling areas, and accessibility features for individuals with disabilities. These barriers were operationalised through specific survey items assessing space design, crowding, waiting areas, and overall accessibility.

Personal barriers were conceptualised as individual characteristics and behaviours of pharmacists that could impede effective patient interactions. This includes aspects such as the pharmacist’s approachability, communication skills, and willingness to address patient inquiries. These barriers were operationalised through survey items evaluating pharmacist confidence, knowledge, and the ease of discussing sensitive health issues. This framework guided the development and assessment of relevant survey domains.

### Inclusion and exclusion criteria

The survey advertisements were thoughtfully constructed to prevent participation from individuals who did not meet the specified criteria. The inclusion criteria required participants to be proficient in either Arabic or English, aged 18 years or older, and residing in the UAE, with voluntary consent to participate. Exclusion criteria included individuals residing outside the UAE, those without proficiency in Arabic or English, individuals under the age of 18, and those unwilling to participate.

### Data collection

Surveys were distributed across various social media platforms, including but not limited to Facebook and Instagram. Prospective participants were initially presented with a comprehensive overview explaining the study's objectives and asked to provide informed consent before completing the questionnaire. Clear assurances regarding confidentiality and anonymity were provided to participants, and a comprehensive participant information page was supplied. It is designed to provide them with a thorough understanding of the study's rationale, methodology, and relevant details.

To ensure data completeness and prevent missing responses, the questionnaire was developed using Google Forms with mandatory response fields. Participants were required to answer each question before proceeding to the next section. If any question was left unanswered, the system prompted participants to return and complete the missing information, thereby ensuring that all submitted responses were complete.

Additionally, measures were implemented to prevent duplicate submissions. Each participant was restricted to a single submission per device, with the system blocking further responses from the same device after the initial submission. This approach minimised the risk of duplicate entries and enhanced the reliability of the data, contributing to a more accurate and robust sampling process.

### Statistical analysis

Statistical analysis was conducted using SPSS: IBM SPSS Statistics (Version 28). Categorical variables were expressed as frequencies and percentages, while continuous variables were presented as medians with corresponding 95% confidence intervals. Cronbach's alpha coefficients were calculated to assess the internal consistency of latent variables. Binary regression models were constructed to explore relationships between sociodemographic characteristics and communication barriers. Independent variables included age, gender, marital status, monthly income, level of education, type of pharmacy for medical advice, age of pharmacists typically interacted with, and pharmacy location. The significance level was set at *p* < 0.05 for all analyses.

## Results

Table 1 summarises the sociodemographic characteristics of the participants enrolled in the study. Approximately one-third of participants were in the age range of 18–24 years (35.71%), with a slightly higher proportion of females (54.93%). Half of the participants reported being single (50%), and the majority identified as Expats Arab (81.03%). Additionally, most participants held a bachelor's degree (69.21%), with 42.12% having a medical background. The predominant language spoken among participants was Arabic (95.07%), although a substantial portion also spoke English (83.5%). Approximately one-third of participants reported having chronic diseases (30.54%).

**Table 1.** Sociodemographic characteristics of participants (*N* = 406).

Variables		Frequency (%)
Age (years)	18–24 years	145 (35.71)
	25–32 years	66 (16.26)
	33–40 years	63 (15.52)
	41–50 years	78 (19.21)
	>50 years	54 (13.3)
Gender	Female	223 (54.93)
	Male	183 (45.07)
Marital status	Single	203 (50)
	Married	172 (42.36)
	Divorced	18 (4.43)
	Widowed	13 (3.2)
Nationality	Expats Arab	329 (81.03)
	Local	47 (11.58)
	Expats non-Arab	30 (7.39)
Level of education	Bachelor	281 (69.21)
	Postgraduate	64 (15.76)
	High School	61 (15.02)
Professional background	Non-medical	235 (57.88)
	Medical	171 (42.12)
Spoken language	Arabic	386 (95.07)
	English	339 (83.5)
	Urdu	12 (2.96)
	Other*	18 (4.43)
Chronic diseases	No	282 (69.46)
	Yes	124 (30.54)

*Bangla, Ukrainian, Kurdish, Russian, French, Tamil, Deutsch (German), Portuguese, Dutch, Turkish, French.

Table 2 presents the demographics of pharmacists and pharmacies involved in the study. A significant portion of the study participants sought medical advice from chain pharmacies (62.07%), with the majority of pharmacies located in Abu Dhabi (60.84%). Most participants reported frequent visits to pharmacies seeking daily medical advice (69.7%) and typically interacted with pharmacists in their 30s (60.1%). Additionally, nearly 40% of participants reported encountering more than five customers in their most frequently visited pharmacy.

**Table 2.** Demographics of pharmacists and pharmacies (*N* = 406).

Variables		Frequency (%)
Type of pharmacy	Chain pharmacy	252 (62.07)
	Independent pharmacy	154 (37.93)
Pharmacy location	Abu Dhabi	247 (60.84)
	Dubai	44 (10.84)
	Alain	37 (9.11)
	Northern Emirates*	78 (19.21)
Number of customers seeking daily advice during the participant’s last visit**	Yes	283 (69.7)
	No	123 (30.3)
Number of customers in the pharmacy during the participant’s last visit***	No one, just me	43 (10.59)
	Less than 5 customers	169 (41.63)
	5–15 customers	118 (29.06)
	16–25 customers	21 (5.17)
	More than 25 customers	23 (5.67)
	Not sure/Don’t know	32 (7.88)
Age of pharmacist whom the participant interacted with during the last visit	20s	52 (12.81)
	30s	244 (60.1)
	40s	82 (20.2)
	50s	19 (4.68)
	More than 50s	9 (2.22)
Frequency of pharmacy visits	Weekly	22 (5.42)
	Monthly	84 (20.69)
	Every 3 months	123 (30.3)
	Every 6 months	35 (8.62)
	Yearly	9 (2.22)
	When I need something	133 (32.76)

*Northern Emirates includes Ajman, Fujairah, Ras Al Khaimah, and Umm Al Quwain.

**Based on the type of pharmacy you recently visited, does the pharmacy have a high number of customers seeking medical advice on a daily basis?

***During the time you spent in the pharmacy at your last visits, how many customers were there to seek their medical advice from the same pharmacy?

**Figure 1.**
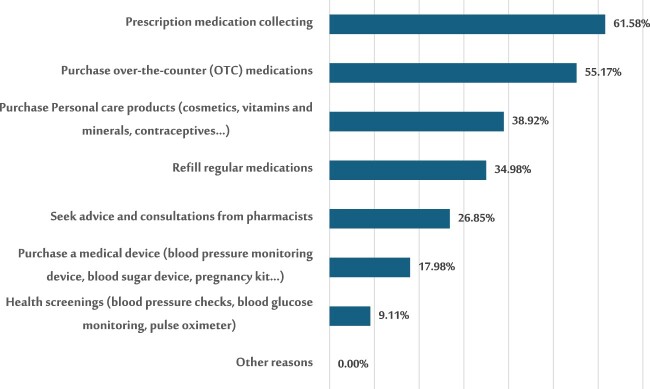
The primary reasons prompting visits to pharmacies.

Figure 1 illustrates the predominant motivations prompting patients’ visits to the pharmacy. The most frequently cited reasons were ‘To collect a prescription medication' and ‘Purchase over-the-counter (OTC) medications’, representing 61.58% and 55.17% of responses, respectively. Conversely, health screening emerged as the least common reason for pharmacy visits, accounting for only 9.11% of responses. Additionally, participants identified other motives, including refilling regular medication, seeking advice and consultations, purchasing personal care products, and acquiring medical devices.

Table 3 summarises the participants’ perceptions regarding factors affecting effective communication within the pharmacy setting. The most cited obstacle to effective communication was identified as ‘The crowded and noisy environment at the prescription area' (61.58%), followed by ‘The busy pharmacist who didn’t give enough counseling time for patients' and ‘Lack or no privacy area (counseling area)' (45.81% and 45.07%, respectively). Conversely, factors perceived as less impactful on communication effectiveness included ‘The pharmacist and the patient use the same language' and Comfortable Pharmacy design and decoration' (28.08% each), along with ‘Comfortable waiting area' (36.21%).

**Table 3.** Physical and environmental barriers affecting effective communication (*N* = 406).

Factors	Frequency (%)
Crowded and noisy environment at the prescription area	250 (61.58)
Busy pharmacist unable to allocate sufficient counseling time for patients	186 (45.81)
Lack or absence of privacy area (counseling area)	183 (45.07)
Height of the prescription counter	176 (43.35)
Comfortable waiting area	147 (36.21)
Comfortable pharmacy design and decoration	114 (28.08)
Language barrier between pharmacist and patient	114 (28.08)
Adequate light, visibility, and visual quality	100 (24.63)
Other*	2 (0.49)

*Responses included the need for pharmacists to handle uneasy patients with good manners and concerns about pharmacist qualifications and knowledge adequacy.

Table 4 illustrates the participants’ perspectives regarding personal barriers. About 40.89% of respondents indicated discomfort in discussing sensitive situations or issues with the pharmacist. However, the majority (74.14%) disagreed or strongly disagreed with the assertion that pharmacists lacked the knowledge to address their inquiries. Furthermore, nearly half of the participants disagreed or strongly disagreed with the statement ‘the pharmacist showed a lack of interest in answering my questions on my last visit'.

**Table 4.** Personal barriers (*N* = 406).

Statement	Strongly Disagree	Disagree	Neutral	Agree	Strongly Agree	Median (IQR)
I feel discomfort in discussing sensitive situations/issues with the pharmacist	21 (5.17%)	82 (20.2%)	137 (33.74%)	120 (29.56%)	46 (11.33%)	3 (2–4)
In my opinion, the pharmacists were not knowledgeable enough to answer my questions	76 (18.72%)	225 (55.42%)	88 (21.67%)	13 (3.2%)	4 (0.99%)	2 (2–3)
In my opinion, the pharmacist showed a lack of interest in answering my questions on my last visit	43 (10.59%)	155 (38.18%)	94 (23.15%)	88 (21.67%)	26 (6.4%)	3 (2–4)

IQR: Interquartile Range.

Table 5 provides insights into the factors influencing participants’ preferences for one pharmacy over another. The primary determinant was the pharmacy's proximity to their location, cited by 75.12% of respondents. Other influential factors included ‘Easy waiting times' and ‘Insurance coverage’, mentioned by 47.29% and 42.86% of participants, respectively. Interestingly, additional services such as blood pressure, glucose, and pulse measurements were among the least influential factors, with only 20.2% of respondents considering them.

**Table 5.** Factors influencing pharmacy preference (*N* = 406).

Reasons*	Frequency (%)
Nearby location	305 (75.12)
Easy waiting times	192 (47.29)
Insurance coverage	174 (42.86)
Personality and knowledge of pharmacist**	172 (42.36)
Pricing and discounts	136 (33.5)
Availability of specific medications	133 (32.76)
Additional services (e.g. blood pressure, glucose)	82 (20.2)

*Respondents could select multiple reasons.

**Trust in a specific pharmacist at a particular branch.

Binary logistic regression analyses were conducted to examine the factors affecting environmental and personal barriers within pharmacy settings. These barriers were categorised into binary variables. Responses were dichotomised, with scores above a predetermined threshold indicating the presence of a barrier, and scores below indicating its absence. This approach allowed for a clear and consistent categorisation of barriers, facilitating their use as independent variables in the logistic regression model. Table 6 presents the results between environmental barriers and related variables. Notable associations were found, including the frequency of pharmacy visits, reasons for visiting the pharmacy, and preferences for certain pharmacy characteristics. Customers who visit the pharmacy more frequently (weekly) exhibit a higher likelihood of belonging to the high environmental barriers in comparison with those who visit the pharmacy only when needed (OR: 5.502, 95% CI: 1.860–16.274, *p* = 0.002). Moreover, the patients with a single reason to visit the pharmacy demonstrate a substantially higher likelihood of belonging to the high environmental barriers in comparison to customers who have three or more reasons to visit (OR: 6.102 95% CI: 3.283–11.343, *p* < 0.001). Additionally, customers who don’t consider the easy waiting time as a factor to prefer one pharmacy to another exhibited increased odds of being in the high environmental barrier group when compared with those who consider it as an influential factor (OR: 2.033, 95% CI: 1.188–3.478, *p* = 0.01). Similarly, customers who do not consider the nearby location as an influential factor exhibit higher odds of belonging to the higher environmental barriers’ groups (OR: 2.111, 95% CI: 1.216–3.663, *p* = 0.008).

**Table 6.** Binary regression analysis between environmental barriers and related variables.

Characteristics		*p*-value	OR	95% C.I. for OR (Lower, Upper)
Type of pharmacy mostly preferred	Independent pharmacy (Ref)		–	–
	Chain pharmacy	0.880	0.959	(0.555, 1.655)
Pharmacy location	Northern Emirates (Ref)		–	–
	Abu Dhabi	0.727	1.130	(0.569, 2.247)
	Dubai	0.491	0.705	(0.260, 1.908)
	Alain	0.481	0.681	(0.234, 1.983)
Frequency of visits	When I need something (Ref)		–	–
	Weekly	**0**.**002**	5.502	(1.860, 16.274)
	Monthly	0.082	1.908	(0.920, 3.958)
	Every three months	0.231	1.512	(0.769, 2.973)
	Every six months	0.609	0.760	(0.266, 2.171)
	Yearly	0.231	2.689	(0.532, 13.591)
Number of reasons for pharmacy visit	Three and more (Ref)		–	–
	One	**< 0.001**	6.102	(3.283, 11.343)
	Two	0.140	1.697	(0.841, 3.424)
Reasons for pharmacy preference: *easy waiting time*	Yes (Ref)		–	–
	No	**0**.**010**	2.033	(1.188, 3.478)
Reasons for pharmacy preference: *nearby location*	Yes (Ref)		–	–
	No	**0**.**008**	2.111	(1.216, 3.663)

Note: Ref represents the reference category and the values in parentheses represent the 95% confidence interval for the odds ratio.

Additionally, Table 7 illustrates the results of sociodemographic characteristics with personal barriers. Significant associations were observed, particularly regarding nationality, pharmacy type preference, frequency of pharmacy visits, and age of the pharmacist interacted with. The analysis shows that the customers who are Expats of Arab nationality showed twice the likelihood of belonging to the high personal barriers in comparison to those of local nationality (OR: 2.54, 95% CI: 1.059–3.981, *p* = 0.033). Furthermore, the results found that customers who primarily sought medication or medical advice from chain pharmacies most of the time were more likely to fall into the personal barrier group when compared with those who preferred independent pharmacies (OR: 1.609, 95% CI: 1.047–2.472, *p* = 0.03). Moreover, the patients who visited the pharmacy every six months demonstrated decreased odds of being in the high personal barrier group when compared with those who visited it when they needed something (OR: 0.333, 95% CI: 0.143–0.774, *p* = 0.011). Additionally, the participants who interacted with pharmacists in their 20s and 30s exhibited increased odds of being in the high personal barrier group when compared with those who interacted with older (OR: 12.357, 95% CI: 1.361–112.173, *p* = 0.025 and OR: 9.906, 95% CI: 1.164–84.304, *p* = 0.036, respectively).

**Table 7.** Binary regression analysis of sociodemographic characteristics with personal barriers.

Characteristics		*p*-value	OR	95% C.I. for OR (Lower, Upper)
Nationality	Local (Ref)		–	–
	Expats Arab	**0**.**033**	2.054	(1.059, 3.981)
	Expats non-Arab	0.208	0.512	(0.181, 1.451)
Type of pharmacy mostly preferred	Independent pharmacy (Ref)		–	–
	Chain pharmacy	**0**.**030**	1.609	(1.047, 2.472)
frequency of visits	When I need something (Ref)		–	–
	Weekly	0.411	0.674	(0.263, 1.727)
	Monthly	0.637	0.870	(0.489, 1.548)
	Every three months	0.482	1.210	(0.711, 2.058)
	Every six months	**0**.**011**	0.333	(0.143, 0.774)
	Yearly	0.642	1.428	(0.317, 6.429)
Age of interacted pharmacist	More than 50s (Ref)		–	–
	20s	**0**.**025**	12.357	(1.361, 112.173)
	30s	**0**.**036**	9.906	(1.164, 84.304)
	40s	0.055	8.382	(0.955, 73.579)
	50s	0.300	3.465	(0.331, 36.282)

## Discussion

The present study highlighted key barriers to effective communication in community pharmacies from the perspective of general public which includes both patients and non-patient visitors to community pharmacies in the UAE. The findings identified significant environmental and personal factors that impede optimal interactions between general public and pharmacists. Recognising these barriers is essential not only for improving patient-centered care and enhancing the overall quality of pharmacy services but also for informing policymakers and guiding improvements in pharmacy practice.

### Environmental barriers

One of the most prominent environmental barriers identified was the crowded and noisy environment at the prescription area, reported by 61.6% of participants. This finding aligns with previous research highlighting how a high-volume setting can negatively impact communication quality by limiting the time and attention pharmacists can dedicate to individual patients (Owens & Baergen, 2021). The absence of privacy areas for counseling was also a significant barrier (45.1%), indicating a need for dedicated spaces where confidential discussions can occur. Privacy is crucial in pharmacy settings to foster open communication, especially regarding sensitive health issues (Hattingh et al., 2016).

Moreover, the study found that participants who frequented pharmacies more often reported higher environmental barriers. This could be attributed to their increased exposure to suboptimal conditions, leading to cumulative dissatisfaction. Frequent visits to pharmacies are often necessitated by chronic conditions, making it even more critical to address these barriers to ensure continuous and effective care. Other identified barriers include busy pharmacists as significant barriers to effective communication. These findings underscore the importance of creating conducive environments within pharmacies to facilitate meaningful interactions between general public and pharmacists. Implementing strategies such as designated counseling areas, soundproofing measures, and adequate staffing levels during peak times could help alleviate these environmental barriers (Ilardo & Speciale, 2020; Odukoya et al., 2014)

The COVID-19 pandemic has significantly increased the workload of community pharmacists, highlighted by surges in prescription renewals, drug shortages, and unremunerated clinical interventions. Despite lacking specific guidance, pharmacists adapted with longer shifts and new scheduling patterns (Gregory & Austin, 2020; Kharaba, Moutraji, et al., 2022; Pantasri, 2022). This trend, observed in the UAE, underscores the need for targeted interventions to support pharmacists in managing these ongoing challenges while maintaining high-quality patient care (Kharaba, Moutraji, et al., 2022).

### Personal barriers

Personal barriers were also significant, with 40.89% of respondents expressing discomfort in discussing sensitive issues with pharmacists. This discomfort can be attributed to various factors, including cultural norms, perceived judgment, and previous negative experiences (Kamal & Jacob, 2023; Tan et al., 2024). The finding that the majority of participants disagreed with the statement that pharmacists lacked knowledge (74.14%) suggests that while pharmacists are perceived as knowledgeable, personal discomfort and relational barriers still impede effective communication.

Interestingly, we found that participants interacting with younger pharmacists (in their 20s and 30s) were more likely to report personal barriers. This could be due to a perceived lack of experience or confidence in younger pharmacists, highlighting the need for enhanced training and support for early-career pharmacists to build general public trust, and a need for improved pharmacist-general public rapport and communication skills (Esmalipour et al., 2021; Ng et al., 2023). However, the majority of respondents expressed confidence in pharmacists’ knowledge and disagreed with the perception of pharmacists lacking interest in addressing general public inquiries. This suggests that efforts should focus on enhancing interpersonal communication skills and building trust between general public and pharmacists, rather than solely on improving pharmacists’ technical knowledge (Gregory & Austin, 2021).

### Factors influencing pharmacy preference

Proximity to the pharmacy's location was the most significant factor influencing pharmacy choice, cited by 75.12% of participants. This emphasises the importance of local community pharmacies in providing accessible healthcare and highlights the importance of accessibility and convenience in shaping general public behaviour (Valliant et al., 2022). However, the preference for chain pharmacies (62.07%) suggests that other factors such as perceived reliability, availability of services, and insurance coverage may also play crucial roles (Gist-Mackey et al., 2024; Shiyanbola & Mort, 2014).

Our results revealed that participants who did not consider easy waiting times and nearby locations as influential roles in general publics’ decision-making processes were more likely to report high environmental barriers. This indicates that logistical convenience is critical to public satisfaction and can mitigate perceived environmental challenges. Understanding these preferences can inform pharmacy management strategies aimed at optimising service delivery and enhancing general public satisfaction. Given the increasing integration of new technologies in pharmacy practice (Jirjees et al., 2022), it is crucial to address communication barriers and the need for comprehensive ethical training to ensure that both pharmacists and publics can use them effectively and responsibly.

Further analyses revealed several associations between sociodemographic characteristics and perceived barriers. For instance, general publics who visited pharmacies more frequently were more likely to perceive environmental barriers, suggesting that the frequency of interactions may exacerbate communication challenges. Additionally, publics who primarily sought medication or advice from chain pharmacies exhibited higher personal barriers, indicating potential differences in service delivery between chain and independent pharmacies. These findings underscore the need for tailored interventions targeting specific public populations and pharmacy types to address communication barriers effectively.

### Implications and recommendations

The findings underscore the need for strategic interventions to enhance communication in community pharmacies. Addressing environmental barriers requires infrastructural changes, such as the implementation of private counseling areas and measures to reduce noise and crowding. Pharmacists should be trained to manage busy environments effectively, ensuring they can provide adequate attention to each public even during peak times.

To overcome personal barriers, cultural competence training for pharmacists is essential. This training should focus on building rapport, demonstrating empathy, and creating a welcoming atmosphere that encourages open dialogue. Additionally, mentorship programmes can help younger pharmacists gain confidence and improve their communication skills.

### Study limitations

The use of convenience sampling may limit the generalizability of the findings to the broader population. Additionally, the study relied on self-reported data, which may be subject to recall bias and social desirability bias. Future research could employ more rigorous sampling methods and longitudinal study designs to further explore the dynamics of pharmacist-patient communication over time. Additionally, qualitative studies could provide deeper insights into general publics’ experiences of communication barriers, allowing for a more focused understanding of the underlying factors.

Moreover, we did not use stratified sampling in our study. Although we aimed to include participants from different regions within the UAE to capture a broad representation of the general public, the lack of stratification means that certain subgroups (e.g. age, gender, or socioeconomic status) may not be fully represented. This is acknowledged as a limitation of the study. Future research may consider stratified sampling to ensure more precise representation of these subgroups. Finally, another potential limitation of this study is the challenge in accurately estimating the exact number of customers in the pharmacy. To address this, we provided options to capture an approximate range, which may affect the precision of the data.

## Conclusion

Effective communication in community pharmacies is paramount to delivering high-quality healthcare. This study highlights significant environmental and personal barriers in the UAE, providing insights into areas needing improvement. By identifying and addressing these barriers, we can develop targeted interventions and training programmes to optimise communication practices, enhance general public satisfaction, and ultimately improve healthcare outcomes. This research serves as a foundation for fostering patient-centered care within community pharmacies, promoting better engagement, trust, and collaboration between general publics and pharmacists. Moreover, the findings offer valuable guidance for policymakers and stakeholders in the healthcare system, facilitating the development of regulations and best practices that will improve communication standards and advance the quality of pharmacy practice.

## Recommendations on policy change

To enhance general public-pharmacist communication, several policy recommendations are proposed. First, mandatory communication skills training for pharmacists should be implemented as part of their continuous professional development to improve the quality of interactions. Additionally, policies should advocate for the design of pharmacies with private counseling areas and accessible layouts, ensuring patient comfort and confidentiality. Regulating pharmacists’ workloads is also essential to prevent burnout and ensure they can devote adequate time to patient communication. Furthermore, policies should focus on improving accessibility for patients with special needs, such as the elderly and those with disabilities, to guarantee equitable access to pharmacy services. Finally, public health campaigns should be launched to raise awareness about the role of pharmacists, encouraging the general public to utilise pharmacy consultation services effectively. These policy changes aim to address communication barriers and improve the overall pharmacy experience for both patients and the general public.

## Supplementary Material

Supplemental Material
