# Supplementary material for: Barriers to effective communication in UAE community pharmacies: general public perspectives on enhancing patient-pharmacist interaction and policy development
Source: J Pharm Policy Pract. 2025 Feb 13;18(1):2460744. doi: 10.1080/20523211.2025.2460744 (PMC11827033; doi:10.1080/20523211.2025.2460744)
Supplement: Supplemental Material [file JPPP_A_2460744_SM0800.docx]

**Supplemental Material**

**(Study Questionnaire)**

**Barriers to communication questioner**

**Section 1. Sociodemographic characteristics of the enrolled participants.**

1. **Age (years):**

- 18-24 years
- 25-32 years
- 33-40 years
- 41-50 years
- more than 50 years

1. **Sex:**

- Male
- Female

1. **Marital status:**

- Married
- Single
- Divorced
- Widowed

1. **Nationality:**

- Expats Arab
- Expats non-Arab
- Local

1. **Level of education:**

- High school
- Bachelor
- Postgraduate (Master / PhD)

1. **Professional background:**

- Medical background
- Non-Medical background

1. **Spoken language (you can select multiple answers):**

- Arabic
- English
- Urdu
- Other

1. **Do you have any chronic diseases?**

- Yes
- No

1. **What is the type of pharmacy you seek to get your medication or seek medical advice MOST OF TIMES?**

- Chain pharmacy
- Independent Pharmacy

1. **Pharmacy location:**

- Abu Dhabi
- Alain
- Dubai
- Northern Emirates (Sharjah, Ajman, Fujairah, Umm Al Quwain)

1. **Based on the type of pharmacy that is visited recently, does the pharmacy have a high number of customers seeking medical advice on a daily basis?**

- Yes
- No

1. **During the time you spent in the pharmacy at your last visits, how many customers were there to seek their medical advice from the same pharmacy?**

- No one, just me
- Less than 5 customers
- 5 - 15 customers
- 16-25 customers
- More than 25 customers
- Not sure/Don’t know

1. **Age of Pharmacist that you in general interact with in your last visit?**

- 20’s
- 30’s
- 40’s
- 50’s
- More than 50’s

1. **How many times do you visit the pharmacy?**

- Weekly
- Monthly
- Every three months
- Every six months
- Yearly
- When I need something

**Section 2: Most Common reasons lead the patients to visit a pharmacy.**

1. **What are the most common reasons that lead you to visit a pharmacy? (you can select multiple answers)**

- Refill regular medications
- To collect a prescription medication
- Purchase over-the-counter (OTC) medications
- Seek advice and consultations from pharmacists
- Purchase Personal care products (cosmetics, vitamins and minerals, contraceptives…)
- Purchase a medical device (blood pressure monitoring device, blood sugar device, pregnancy kit...)
- Health screenings: such as blood pressure checks, blood glucose monitoring, pulse oximeter
- Other reasons

**Section 3: The Physical / Environmental Barriers that affect the effective communication:**

1. **Which factors do you believe can affect or act as barriers to effective communication? (you can select multiple answers)**

- Height of the prescription counter
- The crowded and noisy environment at the prescription area.
- Lack or no privacy area (counseling area)
- Appropriate light, visibility, and visual quality
- Comfortable Pharmacy design and decoration
- Comfortable waiting area
- The busy pharmacist who didn’t give enough counseling time for patients
- The pharmacist and the patient use the same language
- Other

**Section 4: Personal Barriers:**

1. **I feel discomfort in discussing sensitive situations/issues with the pharmacist.**

- Strongly disagree
- Disagree
- Neutral
- Agree
- Strongly agree

1. **In my opinion, the pharmacists were not knowledgeable enough to answer my questions.**

- Strongly disagree
- Disagree
- Neutral
- Agree
- Strongly agree

1. **In my opinion, the pharmacist showed a lack of interest in answering my questions on my last visit:**

- Strongly disagree
- Disagree
- Neutral
- Agree
- Strongly agree

1. **What reasons make you prefer one pharmacy over the other? Tick all that applied.**

- Nearby location
- Personality and knowledge of pharmacist (trust one of the pharmacists in an specific branch)
- Easy Waiting Times
- Additional Services (blood pressure, glucose, pulse measures)
- Insurance Coverage
- Pricing and Discounts
- Availability of specific medications

**Section 5.** **What do you recommend regarding improving the quality of pharmaceutical care communication?**
